# Circadian signatures of anterior hypothalamus in time-restricted feeding

**DOI:** 10.12688/f1000research.125368.1

**Published:** 2022-09-22

**Authors:** Meiyu Zhou, Jianghui Chen, Rongfeng Huang, Haoran Xin, Xiaogen Ma, Lihua Li, Fang Deng, Zhihui Zhang, Min-Dian Li

**Affiliations:** 1 Department of Cardiovascular Medicine, Center for Circadian Metabolism and Cardiovascular Disease, Southwest Hospital, Third Military Medical University (Army Medical University), Chongqing, 400038, China; 2 Department of Pathophysiology, College of High Altitude Military Medicine, Army Medical University, Chongqing, 400038, China

**Keywords:** Circadian rhythm, time-restricted feeding, entrainment, anterior hypothalamus, transcriptomics, Hippo signaling, oxidative phosphorylation, amino acid degradation

## Abstract

**Background:** Meal timing resets circadian clocks in peripheral tissues, such as the liver, in seven days without affecting the phase of the central clock located in the suprachiasmatic nucleus (SCN) of the hypothalamus. Anterior hypothalamus plays an essential role in energy metabolism, circadian rhythm, and stress response. However, it remains to be elucidated whether and how anterior hypothalamus adapts its circadian rhythms to meal timing.

**Methods:** Here, we applied transcriptomics to profile rhythmic transcripts in the anterior hypothalamus of nocturnal female mice subjected to day- (DRF) or night (NRF)-time restricted feeding for seven days.

**Results:** This global profiling identified 128 and 3,518 rhythmic transcripts in DRF and NRF, respectively. NRF entrained diurnal rhythms among 990 biological processes, including ‘Electron transport chain’ and ‘Hippo signaling’ that reached peak time in the late sleep and late active phase, respectively. By contrast, DRF entrained only 20 rhythmic pathways, including ‘Cellular amino acid catabolic process’, all of which were restricted to the late active phase. The rhythmic transcripts found in both DRF and NRF tissues were largely resistant to phase entrainment by meal timing, which were matched to the action of the circadian clock. Remarkably, DRF for 36 days partially reversed the circadian clock compared to NRF.

**Conclusions:** Collectively, our work generates a useful dataset to explore anterior hypothalamic circadian biology and sheds light on potential rhythmic processes influenced by meal timing in the brain (www.circametdb.org.cn).

## Introduction

Meal timing resets circadian clocks in peripheral tissues (peripheral clocks), particularly in the liver. ^1^^–^^3^ Day time-restricted feeding (DRF) in nocturnal rodents is misaligned with daily rhythms generated by the circadian clock. DRF reverses the liver clock within seven days in mice without altering the central clock located in the suprachiasmatic nucleus (SCN). ^4^^,^^5^ DRF contributes to increased adiposity and glucose intolerance when combined with high-fat diet feeding. ^6^^–^^9^ Conversely, night time-restricted feeding (NRF), is known to protect against metabolic diseases, such as obesity, insulin resistance, and fatty liver in rodents. ^10^^,^^11^ Nevertheless, both DRF and NRF increase longevity in mice, when compared to arrhythmic feeding. ^12^

Emerging evidence shows that peripheral clocks entrain to DRF in a tissue-specific manner. ^13^^,^^14^ Peripheral clocks in the heart and kidneys exhibit resistance to phase entrainment by DRF in female mice, so do the diurnal transcriptomes in these organs. ^14^ This effect is also present in male mice. ^13^ Of note, the liver clock seems to condition circadian rhythms in transcriptome in distant organs such as the lungs and adipose tissue. ^13^ The tissue specificity of phase entrainment to DRF lends support to the proposed network organization or hierarchy of peripheral clocks. ^15^^–^^17^

Anterior hypothalamus is a center for circadian rhythm biogenesis, thermal regulation, endocrine functions, and energy metabolism. It includes the medial preoptic nucleus, supraoptic nucleus, SCN, anterior hypothalamic nucleus, and the paraventricular nucleus of the hypothalamus (PVH). While the SCN generates circadian rhythms, the PVH orchestrates circadian rhythms of metabolism and endocrine factors. ^18^ In addition, the PVH is a hub for nutrient sensing and receives neural signaling from the hunger neurons located in the posterior hypothalamus. ^19^^,^^20^ The SCN and PVH host cellular circadian clocks, and the former projects to the latter to output circadian signals. ^21^ Perceivably, the SCN clock is coupled to the light/dark cycle, whereas the PVH clock integrates circadian signals from both light and food. Genome-wide transcript profiling studies have been done in the SCN or the hypothalamus for rhythmic transcripts in constant darkness. ^22^^,^^23^ It remains to be elucidated whether and how anterior hypothalamus adapts its circadian biology to meal timing in mice.

To explore the regulation of anterior hypothalamic circadian biology by time-restricted feeding, we took a systems approach to profile rhythmic transcripts in this tissue from NRF and DRF female mice. We determined rhythmicity features of diurnal transcripts as well as the category and distribution of the enriched rhythmic pathways. This is followed by examining phase entrainment of the circadian clock, rhythmic transcripts and pathways found in both NRF and DRF tissues. Lastly, we compared the profiles of clock genes in the typical seven-day time-restricted feeding regimen to those in the 36-day long-term regimen in order to elucidate the dynamics of the circadian clock. In summary, we have defined circadian signatures of anterior hypothalamus in time-restricted feeding and generated a useful resource to explore the physiology and pathophysiology associated with different types of time-restricted feeding.

## Methods

### Animals

Animal use was approved by the Laboratory Animal Welfare and Ethics Committee of the Third Military Medical University, China (No. AMUWEC20201106, approval date: 2020/04/15). All experiments conform to the relevant regulatory standards of the institution. All efforts were made to ameliorate harm to animals such as daily monitoring of food and water availability and routine husbandry. This study is reported in line with the ARRIVE guidelines. ^41^

Special pathogen-free (SPF) mice were purchased from Hunan SJT Laboratory Animal Co. and housed in a SPF barrier facility. C57BL/6J female mice at seven weeks of age were group housed and entrained to a 12h light:12h dark cycle with free access to normal chow diet and water during acclimation for one week. Animals were randomly assigned in a 1:1 ratio to time-restricted feeding groups and fed for seven or 36 days as previously described. ^24^ The body weight statistics were balanced between groups before subjecting to time-restricted feeding so that there was no significant difference in body weight between experimental groups. DRF permits access to food from zeitgeber time 0 (ZT0, light-on time) to ZT12 (light-off time), and NRF allows food access from ZT12 to ZT0. At the end of time-restricted feeding, mice were euthanized by cervical dislocation, and subjected to tissue collection every 4 h for a complete 24-h cycle. Refer to doi:10.1016/j.xpro.2021.100701 for step-by-step procedures. Anterior hypothalamus was dissected, snap frozen in liquid nitrogen and stored in -80°C (n = 4 per group per time-point).

### RNA extraction and sequencing

Total RNA from mouse anterior hypothalamus was extracted by TRIzol method (Invitrogen). RNA integrity (RIN > 7), purity and concentration were assessed and all samples passed the quality control. A total of 200 ng total RNA per sample was subjected to mRNA purification by poly-T oligo magnetic beads and the 150-bp pair-end RNA sequencing in the MGISEQ2000 (BGISEQ-500, RRID:SCR_017979) platform for a minimal coverage of 43.1 million reads by BGI (Shenzhen, China) as described. ^14^ The RNA sequencing unit was blinded of the grouping information. Raw reads were cleaned *via* SOAPnuke (RRID:SCR_015025) (v1.5.2), mapped to the reference genome (GRCm38.p6) by HISAT2 (RRID:SCR_015530) (v2.0.4), aligned to the reference coding gene set by Bowtie (RRID:SCR_005476) (v2.2.5), and quantified using RSEM (RRID:SCR_013027) (v1.2.12). Raw counts were normalized by trimmed mean of M-values by edgeR (RRID:SCR_012802) (v3.30.3) and converted to Wagner’s transcripts per million (TPM). No sample was excluded (Pearson correlation coefficient per time point per group > 0.8). Transcripts with a TPM < 1 in more than 20% of samples were filtered. Data were then analyzed by MetaCycle (v1.2.0). ^25^ The cutoff p-value for a rhythmic transcript was set to BH.Q-value < 0.05.

### Phase set enrichment analysis

Pathway enrichment analysis was performed on rhythmic genes as described ^14^^,^^26^ (Source file: c5.bp.v7.1.symbols.gmt from https://www.gsea-msigdb.org/gsea/msigdb). Mouse gene nomenclature was converted to human gene nomenclature *via* “Human and Mouse Homology Classes with Sequence information” (curated by http://www.informatics.jax.org). Domain was set from 0 to 24, indicating the minimal and maximal period length is 0 and 24 h, respectively. Minimal and maximal items per set are 10 and 10000, respectively. Kuiper’s test FDR q-value < 0.05 is considered as statistical significance.

### Gene expression analysis

Tissue RNA was isolated using Eastep Super Total RNA Extraction Kit (Promega). Complementary DNA (cDNA) was synthesized using the GoScript Reverse Transcription Mix (Promega). cDNA was amplified and analyzed using iTaq universal SYBR Green Supermix (Bio-Rad) and the Bio-Rad CFX96 Real-Time PCR Detection System (Bio-Rad). PCR protocol: DNA denaturation at 95°C for 3 min; denaturation at 95°C for 10 sec; annealing/extension and plate read at 60°C for 30 sec; 40 cycles of quantitative PCR. Results were normalized to u36B4. Period circadian protein homolog 2 (Per2)-forward (F), ATGCTCGCCATCCACAAGA; Per2-reverse (R), GCGGAATCGAATGGGAGAAT; Nuclear receptor subfamily 1 group D member 1 (Nr1d1)-F, TACATTGGCTCTAGTGGCTCC; Nr1d1-R, CAGTAGGTGATGGTGGGAAGTA; D site-binding protein (Dbp)-F, CGTGGAGGTGCTAATGACCTTT; Dbp-R, CATGGCCTGGAATGCTTGA; u36B4-F, AGATGCAGCAGATCCGCAT; u36B4-R, GTTCTTGCCCATCAGCACC. Experiments were repeated twice with similar results.

### Statistics

Statistical analysis was conducted in R Project for Statistical Computing (RRID:SCR_001905) (v 4.0.2) and RStudio (RRID:SCR_000432) (v 1.2.5033). BH.Q-value < 0.05 (MetaCycle, v1.2.0), Kuiper test FDR q < 0.05 (phase set enrichment analysis), or p < 0.05 (Bonferroni test or circacompare, v0.1.0 ^27^) was considered to be statistically significant. Heatmap of expression profile was visualized by R package: pheatmap (RRID:SCR_016418) (v1.0.12) with row-wise scaling and gene-specific clustering by Euclidean correlation.

## Results

### Global transcript profiling of anterior hypothalamus from time-restricted fed female mice

**Figure 1.**
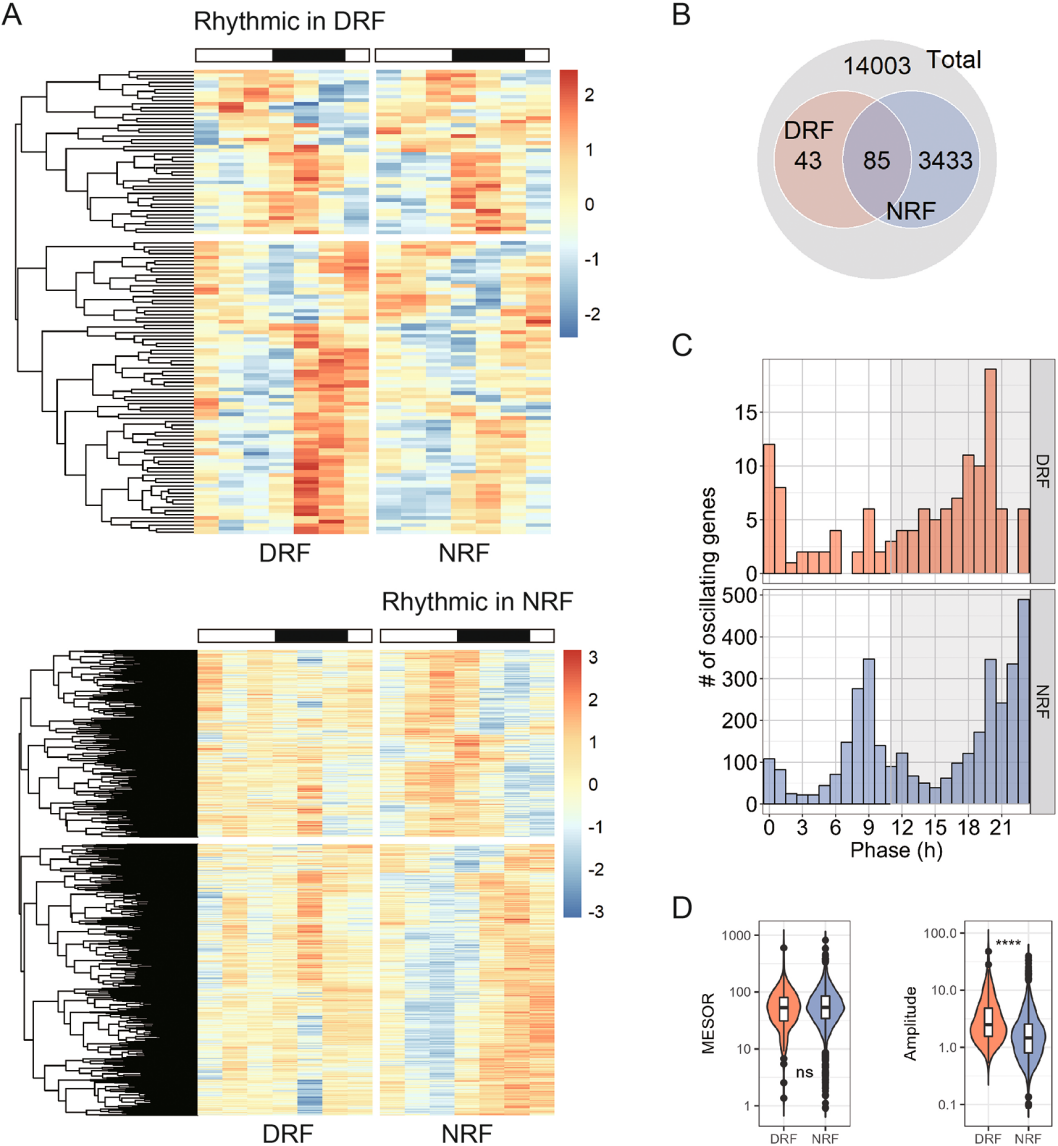
Global transcript profiling of anterior hypothalamus from time-restricted fed female mice. (A) Heatmap showing 24-h expression profiles of rhythmic transcripts in the anterior hypothalamus. Samples were dissected from time-restricted fed nine-week female mice every four hours for a complete daily cycle (n = 4 mice per time point). (B) Interaction of rhythmic transcripts between day time-restricted feeding (DRF) and night time-restricted feeding (NRF). (C) Distribution of the phase. (D) Midline estimating statistic of rhythm (MESOR) and amplitude of rhythmic transcripts. Data are presented as boxplots. Mann-Whitney test; ns, not significant, ****p < 0.0001.

To characterize whether and how diurnal rhythms in the anterior hypothalamus entrain to feeding rhythm, we performed RNA sequencing on samples dissected from nine week-old female C57BL/6J mice that had been subjected to either DRF or NRF for seven days. Seven days of DRF is sufficient to entrain peripheral clocks in female mice. ^14^ Samples were collected every four hours for a complete diurnal cycle and assigned four biological replicates per time point. This is a commonly used study design used in global transcript profiling experiment. RNA sequencing reached a minimal depth of 43.1 million reads, which follows the guideline for genome-scale analysis of circadian rhythms. ^28^ We applied an BH-adjusted p-value (meta2d_BH.Q) of 0.05 to filter rhythmic transcripts and acquired 128 and 3,518 rhythmic transcripts in DRF and NRF tissues, respectively ( Figure 1A). ^41^ Notably, DRF significantly decreased the number of rhythmic transcripts in anterior hypothalamus, compared to NRF. There were 83 rhythmic transcripts that are shared by DRF and NRF tissues ( Figure 1B). As shown in Figure 1C, DRF rhythmic transcripts were clustered in the late active period (ZT18-ZT0), whereas NRF rhythmic transcripts exhibited a bimodal distribution that clustered in the pre-dawn hours (ZT23) and the late sleep phase (ZT9). Averagely speaking, rhythmic transcripts in DRF tissue exhibited a higher amplitude than those in NRF without any significant difference in the basal expression levels (midline estimating statistic of rhythm, MESOR) ( Figure 1D).

### Rhythmic pathways entrained by NRF in the anterior hypothalamus

**Figure 2.**
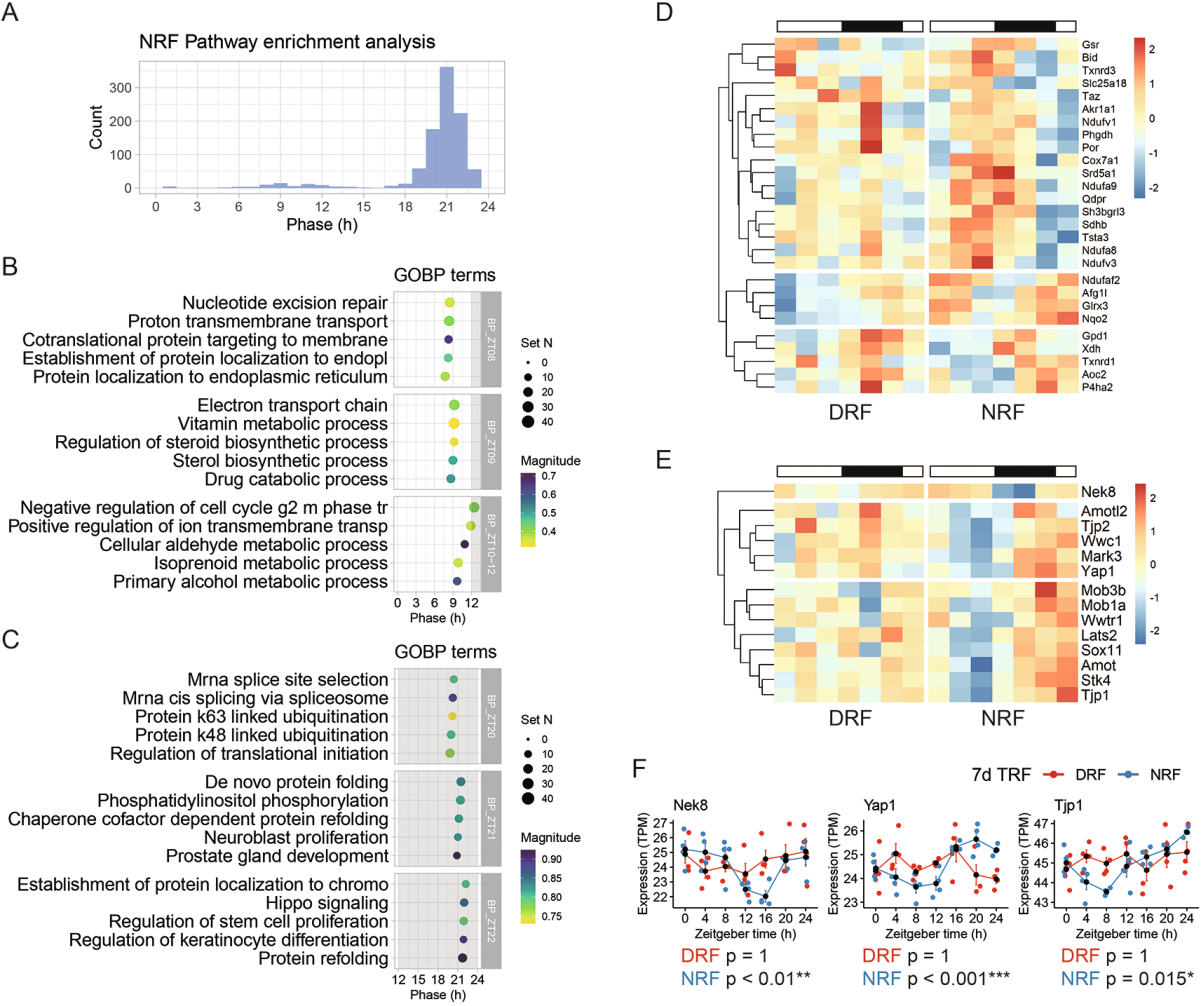
Rhythmic pathways entrained by NRF in the anterior hypothalamus. (A) Phase distribution of enriched rhythmic pathways based on the anterior hypothalamic diurnal transcriptome. (B) Representative rhythmic pathways that peak around zeitgeber time 9 h (ZT9). (C) Representative rhythmic pathways that peak around ZT21. (D) Diurnal expression profiles of the rhythmic genes involved in electron transport chain. (E) Diurnal expression profiles of the rhythmic genes involved in Hippo signaling. (F) Representative rhythmic transcripts related to Hippo signaling. Data are presented as mean ± SEM (n = 4 mice per time point for seven time points). MetaCycle method, *BH adjusted p-value < 0.05, **p < 0.01, ***p < 0.001. NRF, night time-restricted feeding; DRF, day time-restricted feeding; GOBP, gene ontology biological processes; Nek8, NIMA related kinase 8; Yap1, Yes-associated protein 1; Tjp1, Tight junction protein 1.

Next, we performed pathway analysis among rhythmic transcripts found in NRF tissue. As shown in Figure 2A, 990 pathways were significantly enriched and distributed towards the late active phase (ZT21) and the late sleep phase (ZT9). We examined these pathways by hour and found that ‘cotranslational protein targeting to membrane’ (ZT8), ‘electron transport chain’ (ZT9), ‘sterol biosynthetic process’ (ZT9), ‘primary alcohol metabolic process’ (ZT10), and ‘negative regulation of cell cycle g2 m phase tr’ (cell cycle G2-M phase transition) (ZT12) ( Figure 2B). Compared to the late sleep phase, the late active phase is a rush hour for hundreds of rhythmic biological pathways, which are represented by ‘mRNA cis-splicing via spliceosome’ (ZT20), ‘regulation of translational initiation’ (ZT20), ‘protein k63 linked ubiquitination’ (ZT20), ‘protein k48 linked ubiquitination’ (ZT20), ‘de novo protein folding’ (ZT21), ‘phosphatidylinositol phosphorylation’ (ZT21), and ‘Hippo signaling’ (ZT22) ( Figure 2C).

Previously, microarray-based transcriptome profiling of the SCN has shown that the oxidative phosphorylation pathway exhibited circadian rhythm in *ad libitum* feeding and peaked in the late subjective night. ^23^ Examination of the electron transport chain pathway revealed that transcripts related to three respiratory complexes (CI, CII, and CIV) are rhythmic in the NRF anterior hypothalamus and peak in the late active phase ( Figure 2D). For example, these transcripts include NADH dehydrogenase [ubiquinone] flavoprotein 1, mitochondrial ( *Ndufv1*) (CI), NADH dehydrogenase [ubiquinone] 1 alpha subcomplex subunit 9, mitochondrial ( *Ndufa9*) (CI), Succinate dehydrogenase [ubiquinone] iron-sulfur subunit, mitochondrial ( *Sdhb*) (CII), and *Cox7a1* (CIV).

Hippo signaling is essential in organ size control through inhibiting cell proliferation and regulating apoptosis. ^29^ We found that key components from the constituents and regulators of Hippo signaling ( *e.g.*, Transcriptional coactivator YAP1 ( *Yap1*), Serine/threonine-protein kinase LATS2 ( *Lats2*) and *Wwc1*) ( Figure 2E). As shown in Figure 2F, representative transcripts encoding Serine/threonine-protein kinase Nek8 ( *Nek8*), *Yap1,* and *Tjp1* exhibited robust daily rhythm in NRF but lost the rhythmicity upon DRF.

### Rhythmic pathways entrained by DRF in the anterior hypothalamus

**Figure 3.**
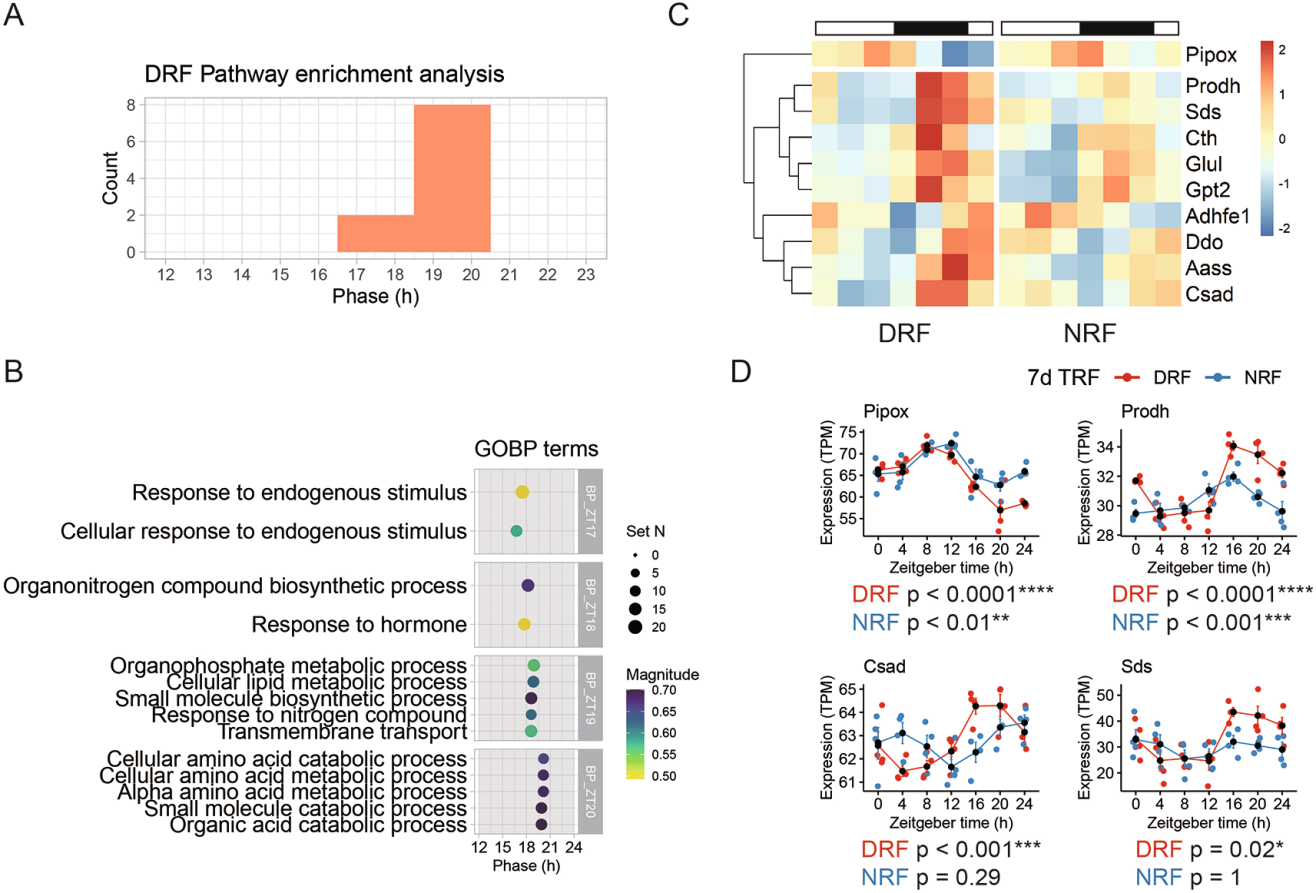
Rhythmic pathways entrained by DRF in the anterior hypothalamus. (A) Phase distribution of enriched rhythmic pathways based on the anterior hypothalamic diurnal transcriptome. (B) Representative rhythmic pathways that peak around zeitgeber time 18.5 h (ZT18.5). (C) Diurnal expression profiles of the rhythmic genes involved in cellular amino acid metabolism. (D) Representative rhythmic transcripts related to cellular amino acid metabolism. Data are presented as mean ± SEM (n = 4 mice per time point for seven time points). MetaCycle method, *BH adjusted p-value < 0.05, **p < 0.01, ***p < 0.001, ****p < 0.0001. NRF, night time-restricted feeding **;** DRF, day time-restricted feeding; GOBP, gene ontology biological processes; Pipox, Pipecolic acid and sarcosine oxidase; Prodh, Proline dehydrogenase 1; Csad, Cysteine sulfinic acid decarboxylase; Sds, Serine dehydrastase.

As shown in Figure 1B, the number of rhythmic transcripts was reduced notably by DRF in the tissue, compared to NRF. Pathway analysis of the 128 rhythmic transcripts revealed 20 enriched rhythmic pathways with peak hours ranging from ZT17 to ZT20 ( Figure 3A). These DRF rhythmic pathways include ‘response to endogenous stimuli’ (ZT17), ‘response to hormone’ (ZT18), ‘organophosphate metabolic process’ (ZT19), and ‘cellular amino acid metabolic process’ (ZT20) ( Figure 3B). With respect to the magnitude of enrichment, amino acid metabolism is highly scored. Hierarchical analysis identified two clusters of rhythmic transcripts involved in cellular amino acid metabolism ( Figure 3C).

*Pipox* transcript encoding pipecolic acid and sarcosine oxidase, an essential enzyme in lysine degradation-forms one cluster by itself, which peaked in the late sleep phase ( Figure 3D). Proline dehydrogenase 1 ( *Prodh*) transcript encodes the first enzyme in proline degradation. Cysteine sulfinic acid decarboxylase ( *Csad*) is an enzyme in cysteine and methionine degradation. Serine dehydrastase ( *Sds*) catalyzes serine and threonine degradation. As shown in Figure 3D, transcripts of *Prodh, Csad,* and *Sds* exhibited robust diurnal oscillation in anterior hypothalamus of DRF mice. In particular, *Csad* and *Sds* did not oscillate in NRF. Thus, transcript signatures suggest that DRF entrains diurnal rhythms of cellular amino acid degradation.

### Food entrainment of the circadian clock in the anterior hypothalamus

**Figure 4.**
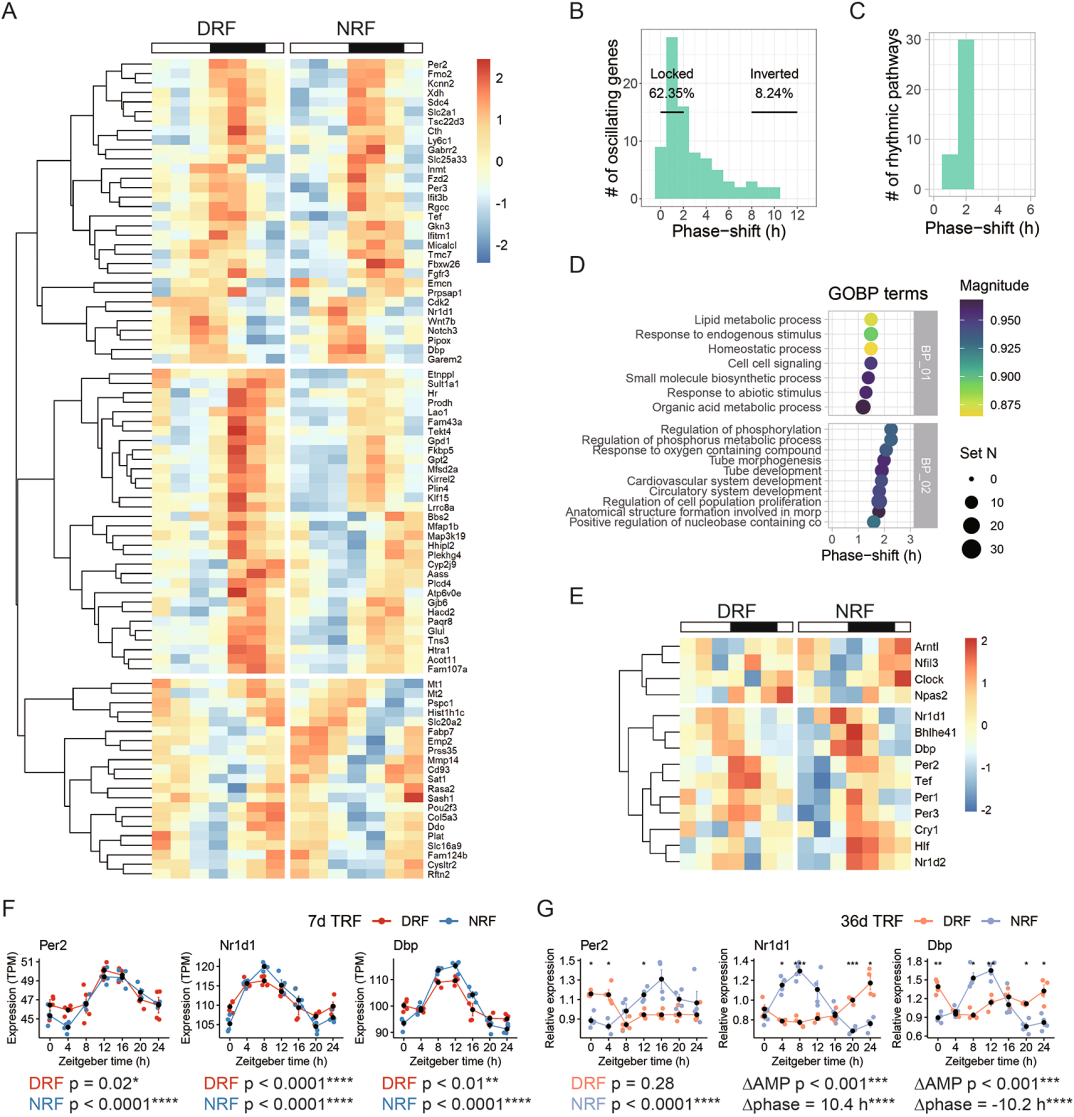
Food entrainment of the circadian clock in the anterior hypothalamus. (A) Hierarchical clustering analysis of rhythmic transcripts found in both DRF and NRF anterior hypothalamus. (B-C) Distribution of the phase-shift between DRF and NRF among (B) rhythmic transcripts or (C) enriched rhythmic pathways. (D) Representative enriched pathways based on shared rhythmic transcripts between DRF and NRF. (E) Hierarchical clustering analysis of the circadian clock genes. (F-G) Diurnal expression of selected clock genes in the anterior hypothalamus from female mice subjected to DRF or NRF for (F) seven days or (G) 36 days. Data are presented as mean ± SEM (n = 4 mice per time point). MetaCycle, *BH adjusted p-value < 0.05; Circacompare method, *p < 0.05, **p < 0.01, ***p < 0.001, ****p < 0.0001. NRF, night time-restricted feeding **;** DRF, day time-restricted feeding; GOBP, gene ontology biological processes; Per2, Period circadian protein homolog 2; Nr1d1, Nuclear receptor subfamily 1 group D member 1; Dbp, D site-binding protein.

After examining circadian signatures of the anterior hypothalamus in each time-restricted feeding regimen, we determined the features associated with rhythmic transcripts found in both NRF and DRF tissues. Hierarchical analysis segregated the 83 dual-cycling transcripts into three clusters ( Figure 4A). DRF reserved only 8.24% of rhythmic transcripts for more than 8 h compared to NRF, whereas more than 60% of the rhythmic transcripts remained phase-locked to the light/dark cycle ( Figure 4B). This is consistent with the results of pathway analysis. In fact, none of the 37 enriched rhythmic pathways were shifted in phase for more than 2 h by DRF compared to NRF ( Figure 4C). Representative enriched pathways include ‘lipid metabolic process’ (phase-shift 1 h), ‘regulation of phosphorylation’ (phase-shift 2 h), and ‘circulatory system development’ (phase shift 2 h) ( Figure 4D).

The weak response in phase entrainment is matched with the response of the circadian clock. Hierarchical analysis segregated the core clock genes into two clusters. One cluster of genes, *e.g.*, Brain-and-muscle ANRT-like 1 ( *Bmal1/Arntl*) and Circadian locomotor output cycles protein kaput ( *Clock*), reached the peak time in the dawn under NRF and damped under DRF ( Figure 4E). The other cluster of genes, *e.g.*, *Per1*, *Per2*, *Per3*, *Nr1d1* and *Nr1d2*, reached the peak time in the dusk in both DRF and NRF ( Figure 4E). As expected, representative transcripts encoding *Per2*, *Nr1d1*, and *Dbp,* was not shifted in phase by DRF ( Figure 4F). After an extension of the time from seven days to 36 days, *Per2* rhythm damped in DRF ( Figure 4G). Remarkably, 36 days of DRF significantly inverted the phase and reduced the amplitude of *Nr1d1* and *Dbp* in the anterior hypothalamus compared to NRF ( Figure 4G).

## Discussion/conclusions

In this study, we applied an unbiased approach to characterize circadian signatures of the anterior hypothalamus in time-restricted fed female mice. We found that DRF, the feeding rhythm that is mis-aligned with the circadian clock, decreased the number of rhythmic transcripts from 3,518 to only 128 compared to NRF within seven days. Pathway analysis identified synchronized rhythms among 990 and 20 biological processes in NRF and DRF, respectively. Typical NRF active pathways include ‘electron transport chain’ (peak time ZT9) and ‘Hippo signaling’ (ZT22). Meanwhile, DRF entrained robust diurnal oscillation among 43 transcripts including those related to ‘cellular amino acid catabolic process’ (ZT20). At the transcriptomic level, only 8.24% rhythmic transcripts were reversed in phase by DRF compared to NRF. This is matched with the resistance of the anterior hypothalamic clock to DRF. However, long-term DRF inverted the phase in some clock genes, such as *Nr1d1* and *Dbp.*

Global profiling of rhythmic transcripts and metabolites have been conducted in a few brain regions particularly including the SCN. In constant darkness, 365 and 642 rhythmic transcripts were identified in the SCN and hypothalamus, respectively. ^22^^,^^23^ High-fat diet feeding increases the number of rhythmic metabolites in the SCN but not in the prefrontal cortex. ^30^ Our transcriptome profiling of anterior hypothalamus uncovered 3,518 rhythmic transcripts in NRF, which suggests that synchronized rhythms from the light and food may markedly increase the prevalence of diurnal rhythms in the hypothalamus.

Anterior hypothalamus is composed of different hub neural regions, such as the preoptic area, SCN and PVH. Our study identified distinct sets of circadian signatures in this tissue under different regimens of time-restricted feeding. Compared to previous studies elucidating the landscape of circadian rhythms in the SCN, hunger neurons, or the hypothalamus, ^22^^,^^23^^,^^31^ NRF entrains diurnal rhythms among a similar set of biological pathways, including oxidative phosphorylation and intracellular trafficking among organelles. By contrast, DRF almost ablates the diurnal rhythms in anterior hypothalamus. This effect may come as a trade-off of two reverse rhythms originating from the light/dark cycles to the SCN and from the feed/fast cycle to the PVH, respectively. Cellular amino acid metabolism is a key circadian signature associated with DRF, which implies the robust daily activity and signaling related to amino acids in anterior hypothalamus. This is supported by a recent study reporting the role of tryptophan metabolism in the entrainment of the brain clock after integrating signals from the light and food. ^32^

The PVH is emerging as a critical peripheral oscillator on top of its well-recognized role as a hub for nutrient sensing and energy homeostasis. ^19^^,^^20^ It outputs circadian rhythm of energy expenditure, the rhythmicity of which is crucial for body weight homeostasis. ^33^ PVH also mediates the circadian tone from the SCN to orchestrate daily rhythm of plasma glucose. ^34^ Recently, it has been shown that corticotrophin-releasing hormone-producing neurons in the PVH receive circadian inputs from the SCN and drive daily release of glucocorticoids. ^35^ Previously, olfactory bulb and cerebellum are proposed as food entrainable peripheral oscillators and involved in food anticipatory behavior induced by DRF. ^36^^–^^38^ In our study, the anterior hypothalamus is partially food-entrainable as indicated by transcript rhythms of *Nr1d1* and *Dbp* after a 36-day DRF. It could be due to the actions in the non-SCN brain area within the anterior hypothalamus, or the SCN. It has been shown recently that time-restricted feeding near the light-on time modulates the SCN functions and impacts thermal homeostasis, locomotor activity and wakefulness. ^39^^,^^40^ These results suggest that brain regions within the anterior hypothalamus may entrain to the feeding rhythm after a long DRF.

In summary, we have determined circadian signatures of the anterior hypothalamus in time-restricted fed mice. The accessibility of the global transcript profiling data in CircaMetDB ^42^^,^^43^ would provide a useful resource to study the physiological significance and the entrainment of circadian rhythms in anterior hypothalamus.

## Data availability

### Reporting guidelines and underlying data

Mendeley Data: F1000Research-125368, Zhou, Chen. https://doi.org/10.17632/h4bvgm2z6s.1. ^41^

This project contains the following underlying data:

- Raw data from RT-qPCR experiment, associated with Figure 4G
- ARRIVE checklist – The ARRIVE Essential 10
- Gene expression profile.csv

Data are available under the terms of the Creative Commons Attribution 4.0 International license (CC-BY 4.0).

The accession numbers for the global profiling dataset:

Gene Expression Omnibus: Global circadian transcript profile of mouse anterior hypothalamus entrained by inverted feeding. Accession number GSE150958; https://identifiers.org/geo:GSE150958. ^42^

China National GeneBank DataBase: Global circadian transcript profile of mouse anterior hypothalamus entrained by inverted feeding. Accession number CNP0001601; https://db.cngb.org/search/project/CNP0001601/. ^43^
