# Supplementary material for: Circadian signatures of anterior hypothalamus in time-restricted feeding
Source: F1000Res. 2022 Sep 22;11:1087. [Version 1] doi: 10.12688/f1000research.125368.1 (PMC9727316; doi:10.12688/f1000research.125368.1)
Supplement: Peer review history

# Peer review: Reviewer response for version 1

**DOI:** 10.5256/f1000research.137666.r154353

**Published:** 2022-12-06

**Authors:** Christopher S. Colwell

**Affiliations:** 1 Department of Psychiatry and Biobehavioral Sciences, University of California Los Angeles, Los Angeles, CA, USA

In this study, the authors used transcriptomics to profile rhythmic transcripts in the anterior hypothalamus of nocturnal female mice subjected to time-restricted feeding that was aligned (night-restricted feeding, NRF) or misaligned (day-restricted feeding, DRF) for seven days. 

 They found 3,518 diurnally rhythmic transcripts in the aligned feeding group while only 128 diurnally rhythmic transcripts in the misaligned feeding group. Impacted pathways were identified.  

**Strengths**:

- RNA-seq appears to be technically solid.
- The authors appear to be well-versed in circadian rhythms and collected samples of the anterior hypothalamus every 4 hrs with n=4 per sample. This sampling frequency is sufficient to look at diurnal rhythms in transcriptions under the aligned (NRF) and misaligned (DRF) time-restricted feeding. 
- An unbiased hierarchical clustering analysis was used. 
- The dramatic loss of rhythmicity from the misaligned feeding group is a surprising finding.   
- The dataset has been deposited and this work may well guide future studies. 

**Weaknesses**:

- The anterior hypothalamus is an anatomically heterogeneous region containing structures central to circadian rhythms like the SCN but also those involved with response to feeding like the PVH. 
- The authors just used female mice so sex differences cannot be explored. 
- Bulk RNA-seq used in this study captures the expression profiles averaged from thousands of cells and single-cell RNA-seq may have been more informative. 
- Control data from mice on *ad lib* feeding and subjected to the same analysis would have added to the study. 
- The transcription factors driving the robust rhythms under aligned feeding and those compromised by the misaligned feeding were not explored. 

Is the work clearly and accurately presented and does it cite the current literature?

Yes

If applicable, is the statistical analysis and its interpretation appropriate?

Yes

Are all the source data underlying the results available to ensure full reproducibility?

Yes

Is the study design appropriate and is the work technically sound?

Yes

Are the conclusions drawn adequately supported by the results?

Partly

Are sufficient details of methods and analysis provided to allow replication by others?

Yes

Reviewer Expertise:

Circadian rhythms, diseases of the nervous system. Time restricted feeding.

I confirm that I have read this submission and believe that I have an appropriate level of expertise to confirm that it is of an acceptable scientific standard.

# Peer review: Reviewer response for version 1

**DOI:** 10.5256/f1000research.137666.r151170

**Published:** 2022-10-12

**Authors:** Jeff R. Jones

**Affiliations:** 1 Department of Biology, Texas A&M University, College Station, TX, USA

This manuscript by Zhou and Chen, *et al.*, focuses on an important question in circadian biology and metabolism: how do clocks in the hypothalamus adjust to meal timing? To address this question, the authors use transcriptomics on anterior hypothalamus tissue taken from female mice every 4 h throughout the day. These mice were subjected to either day-restricted or night-restricted feeding. The authors found that there were many more cycling transcripts in the anterior hypothalamus from mice under night-restricted feeding compared to those under-day restricted feeding. Using pathway enrichment analysis, the authors found that many more rhythmic pathways were entrained in mice under night-restricted feeding compared to mice under day-restricted feeding. Night-restricted feeding-entrained pathways included those involved in oxidative phosphorylation and cell proliferation/apoptosis, and day-restricted feeding-entrained pathways included those involved in amino acid metabolism. Pathways associated with transcripts that cycled in both day-restricted and night-restricted feeding paradigms were largely resistant to food entrainment. Finally, and most excitingly, the authors find that extending day-restricted feeding from 7 d to 36 d inverts the phase of several core clock genes in the anterior hypothalamus. Overall, this study is novel and interesting. I greatly appreciate the online resource the authors developed. I do have a few minor concerns:

1. My largest concern is that the amount of tissue obtained for analysis is large and contains several brain regions including the SCN. Clock gene expression rhythms that are out of phase to those in the SCN have been reported in several of these regions (see, for instance, Figure 1 in Paul *et al.*, *Eur J Neurosci* 2019 ^1^). The authors mention that antiphase rhythms in the SCN and PVN may contribute to the blunting of diurnal rhythms in the anterior hypothalamus observed in mice under the day-restricted feeding paradigm. The authors should elaborate more on this limitation to their study in the Discussion section.
2. The authors should elaborate more on their pathway enrichment analysis findings in the Discussion. Why might different feeding paradigms entrain different metabolic pathways? The authors mention that oxidative phosphorylation has been previously demonstrated to be rhythmic, but what about Hippo signaling (or apoptosis, cell proliferation, etc.)? Why might feeding schedule entrain certain pathways such that they peak at different times of day (ZT 9 vs. ZT 21)? Why are pathways presumably unrelated to metabolism (e.g., prostate gland development) entrained by night-restricted feeding?
3. Was the phase of any other transcript rhythm (including transcripts that aren’t core clock genes) inverted by 36 d of time-restricted feeding compared to 7 d?
4. It would be helpful for the authors to invert the colors on their heatmaps in Figs 2B, C, 3B, 4D. Typically “warmer” colors indicate a higher magnitude response.
5. In the Methods, the authors mention that for phase set enrichment analysis, they set domain from 0 to 24 to indicate that minimal and maximal period lengths were between 0 and 24 h. Is there a reason the authors didn’t examine periods that were longer than 24 h?
6. Was there a reason the authors used female mice instead of a mix of male and female mice?
7. " *We examined these pathways by hour and found that… (Figure 2B)*” and “ *We found that key components from the constituents… (Figure 2E)*” are sentence fragments.

Is the work clearly and accurately presented and does it cite the current literature?

Yes

If applicable, is the statistical analysis and its interpretation appropriate?

Yes

Are all the source data underlying the results available to ensure full reproducibility?

Yes

Is the study design appropriate and is the work technically sound?

Yes

Are the conclusions drawn adequately supported by the results?

Partly

Are sufficient details of methods and analysis provided to allow replication by others?

Yes

Reviewer Expertise:

Circadian biology

I confirm that I have read this submission and believe that I have an appropriate level of expertise to confirm that it is of an acceptable scientific standard.

## References

[1] : Circadian regulation of membrane physiology in neural oscillators throughout the brain. *Eur J Neurosci* .51(1) : 10.1111/ejn.14343 109-138 10.1111/ejn.14343 30633846PMC6625955
